# How to get cheap clean air? Implementation of an affordable high-efficient air filtration ventilation system for operating theatres in a low-resource setting

**DOI:** 10.3205/dgkh000615

**Published:** 2026-01-12

**Authors:** Christian Leonhard Doll, Rolf Opalka, Leonel Tchamo Nguifo, Alaric Tamuedjoun Talom, Lazare Kuate Kamdem, Alexandra Will, Lilith Johannsen, Florian Geiger, Andrej Trampuz

**Affiliations:** 1 Spine and Scoliosis Center, Hessing Foundation, Augsburg, Germany; 2 Mbouo Protestant Hospital, Mbouo/Bandjoun, Cameroon; 3 Faculty of Sciences and Technology, Evangelical University Institute of Cameroon, Mbouo/Bafoussam, Cameroon; 4 cl3an air consulting, Herten, Germany; 5 Foumban Njissé Protestant Hospital, Foumban, Cameroon; 6 Systemic Centre Augsburg, Augsburg, Germany; 7 PRO-IMPLANT Foundation, Berlin, Germany; 8 Charité – Universitätsmedizin Berlin, Corporate Member of Universität Berlin, Humboldt-Universität zu Berlin, and Berlin Institute of Health, Center for Musculoskeletal Surgery (CMSC), Berlin, Germany; 9 Queensland University of Technology, Brisbane, Australia

**Keywords:** operating rooms, ventilation, air conditioning, surgical wound infection, Africa, Cameroon

## Abstract

**Aim:**

Clean air in the operating theatre (OT) is crucial for safe surgery in order to prevent surgical site infections. Air filtration devices are standard in most OT as required by international guidelines and regulations. However, in low-resource settings, common air filtration devices are only scarcely found in OT, mainly due to high cost. Context-adapted low-cost solutions for clean air in OT are urgently needed but not available. The aim of this study was to assess the effectiveness of a new mechanical air filtration system for OTs, to describe its technical details and compare its performance with DIN/ISO standards and natural window ventilation, at a district hospital in Mbouo, Cameroon.

**Methods:**

An affordable (3,500 €) high-efficient air filtration system was developed, locally constructed and implemented. An evaluation was done after 4 years of regular use, comparing the system with ISO/DIN guidelines.

**Results:**

The device was still working properly after 4 years of regular use without any maintenance. The air filtration system reduced particles in the OT by >97%. The microbial contamination in the OT air was reduced by 72% after starting the device. Although it is not fully compliant with all DIN/ISO requirements, the ventilation system greatly improved the cleanliness of the OT air compared to ‘natural’ window-based ventilation.

**Conclusions:**

This pilot study showed the feasibility and quality of a new context-adapted affordable air filtration device for OT in low-resource settings. Further studies and upscaling are needed. Construction plans are freely available.

## Zusammenfassung

**Ziel:**

Saubere Luft im Operationssaal (OP) ist für eine sichere Operation von entscheidender Bedeutung, um postoperative Wundinfektionen zu vermeiden. Luftfiltergeräte sind in den meisten OPs gemäß internationalen Richtlinien und Vorschriften Standard. In ressourcenarmen Umgebungen sind gängige Luftfiltergeräte jedoch nur selten in OPs zu finden, was hauptsächlich auf die hohen Kosten zurückzuführen ist. Es besteht ein dringender Bedarf an kontextangepassten, kostengünstigen Lösungen für saubere Luft im OP, die jedoch nicht verfügbar sind. Ziel dieser Studie war es, die Wirksamkeit eines neuen mechanischen Luftfiltersystems für OPs zu bewerten, seine technischen Details zu beschreiben und seine Leistung mit DIN/ISO-Normen und natürlicher Fensterbelüftung in einem Bezirkskrankenhaus in Mbouo, Kamerun, zu vergleichen.

**Methoden:**

Ein kostengünstiges (3.500 €) hocheffizientes Luftfiltersystem wurde entwickelt, vor Ort gebaut und implementiert. Nach vier Jahren regelmäßiger Nutzung wurde eine Bewertung durchgeführt, bei der das System mit den ISO/DIN-Richtlinien verglichen wurde.

**Ergebnisse:**

Das Gerät funktionierte nach vier Jahren regelmäßiger Nutzung ohne Wartung immer noch einwandfrei. Das Luftfiltersystem reduzierte die Partikel im OP um >97 %. Die mikrobielle Kontamination der OP-Luft wurde nach Inbetriebnahme des Geräts um 72 % reduziert. Obwohl es nicht alle DIN/ISO-Anforderungen vollständig erfüllt, verbesserte das Belüftungssystem die Reinheit der OP-Luft im Vergleich zur „natürlichen” Fensterbelüftung erheblich.

**Schlussfolgerungen:**

Diese Pilotstudie zeigte die Machbarkeit und Qualität eines neuen, kontext-angepassten, erschwinglichen Luftfiltergeräts für Operationssäle in ressourcenarmen Umgebungen. Weitere Studien und eine Skalierung sind erforderlich. Die Baupläne sind frei verfügbar.

## Introduction

Surgical site infections (SSIs) pose a significant threat to patients’ health worldwide, particularly in low- and middle-income countries, where they present a particularly challenging issue [1]. They often result in increased costs, prolonged hospital stays and disability. SSIs may affect up to one third of patients undergoing surgery in Africa [2]. Therefore, preventing SSI is of the utmost importance. The World Health Organization published international guidelines for preventing SSIs [3]. One such measure is the use of mechanical air filtration in operating theatres (OTs), which is the most common method of providing clean air in OTs in well-resourced countries [3]. 

Previous studies have found an association between improved OT ventilation and a reduction in SSIs [4], and have suggested that seasonal air contamination significantly impacts SSI rates in low-resource settings [5]. However, there is a lack of evidence and research on the effect of different air treatment systems, such as mechanical filtration, fans/cooling and natural ventilation, on SSI, particularly in low-resource settings [3].

The two main systems for mechanical filtration are the turbulent mixing airflow ventilation and the laminar airflow ventilation [6]. These systems that are “standard” in OT in high-income countries are expensive, often exceeding 50,000 € installation cost [7] and 13,000 € annual operating cost per year [8]. This high cost may be an important reason why mechanical air filtration systems are not common standard in OT of low and middle income countries [8], [9]. Due to budget restraints, hospitals in low resource settings may prefer simple air conditioning systems not appropriate for use in OT [9] or just window-based ‘natural’ ventilation, which leads to high bacterial burden in the OT air [10]. 

Other possible air filtration alternatives for these contexts may be air sterilisation systems using ultraviolet light or electrostatic adsorption [8], but these systems bear disadvantages in case of power failures: Only filters provide a permanent barrier. Mobile three-stage sterile ventilation units [11] may be another alternative; for example, a mobile three-stage sterile ventilation units costs €21,000, and additional equipment for fresh air supply via HEPA filter 14 with generation of positive pressure in the room costs additional €8,000. Except for the mobile three-stage sterile ventilation unit, the alternatives have the common disadvantage of being not simple and not fail-safe, with possible short service intervals.

Affordable solutions for clean air in OT adapted to the context of low resource settings are therefore urgently needed. This pilot study is, to our knowledge after extensive PubMed and internet search, the first of its kind studying the effectiveness of the developed and implemented low-cost high-efficient context-adapted mechanical air filtration system for OT in a low resource setting.

There are several international and national standards to consider when designing an OT mechanical air ventilation system. To our knowledge, no national norms on OT air filtration exist for Cameroon, the study country. This situation may be similar in many other low- and middle-income countries, in contrast to high-income countries. For Germany, a high-income country, many norms must be considered [12]; the most important of these are used in this study and are stated below. 

DIN 1946-4 is a specific part of the German DIN 1946 standard that focuses on ventilation and air conditioning systems in hospitals and healthcare facilities [13]. It specifies the requirements for operating rooms of different air cleanliness classes. Class I rooms are operating rooms with either laminar airflow (class Ia) or turbulent mixing airflow (class Ib). Class II rooms require lesser standards. 

DIN EN ISO 14644 is an international standard that specifies the classification of air cleanliness in cleanrooms and associated controlled environments [14]. The clean room classes define limits for particle count in the air, with the highest cleanliness in class 1 and the lowest in class 9. 

VDI 6022 is a German guideline that establishes the hygienic requirements for ventilation and air conditioning systems [15]. DIN EN ISO 16890 is an international standard that classifies air filters for ventilation based on their efficiency in removing particles [16]. EN 1822 is a European standard that specifies the classification, testing and performance requirements for high-efficiency particulate air (HEPA) filters and others [17]. In a common 3-stage air filtration for DIN 1946-4 class Ia/b OT, a ISO 16890 ePM1 55% filter (formerly F7) is followed by a ePM1 80% filter (formerly F9) and a EN 1822 H13 HEPA filter, reaching ISO 14644-1 class 5 air cleanliness.

The primary objective of the study is to evaluate the effectiveness of the developed and implemented mechanical air filtration system for OT at the Mbouo Protestant Hospital. The secondary objective is to describe the technical details of this system and its robustness and to compare it to DIN/ISO standards and to window-based ‘natural’ ventilation.

## Methods

### Study design, study site and data collection

This quasi-experimental pilot field study combines a retrospective (technical data) and prospective (microbial data) design to evaluate a novel context-adapted air filtration system. The technical parameters were extracted from the maintenance documents of November 2023. The microbial samples in the OT were collected in May 2024. The study compares these variables with the ventilation system at rest (using ‘natural ventilation’) against activated ventilation system.

The air filtration system was installed in the OT of the Mbouo Protestant Hospital (Hôpital Protestant de Mbouo) in June 2019. This denominational non-profit district-level hospital provides 200 beds and is part of the health department of the Evangelical Church of Cameroon (EEC). It is situated in the West region of Cameroon at about 1,500 m altitude near Bafoussam. About 500 major and 600 minor surgical procedures are performed every year. Before the installation of the air filtrations system, the OT used window-based ventilation, which is quite common in OT in Cameroonian hospitals, besides wall-mounted air conditioning systems.

The air filtrations system has been in regular use for 4 years before the first maintenance was performed in November 2023. COVID travel restrictions and locally unavailable specific measurement devices caused this delay. This maintenance and technical measurement took place in November 2023.

The technical parameters that were extracted from the maintenance documents are: particle counts, temperature, humidity, air velocity and pressure differences at various measurement points inside and outside the OT as well as inside the air filtration device. The following measurement devices were used:

- Differential pressure gauge MPM-10 (PCE Instruments, Germany) with measuring range ±5,000 Pa and accuracy ±0.3% of measuring range;
- Anemometer impeller UT363S (Uni-Trend, China) with measuring range 0.4 to 30m/s and accuracy ±(5%+0.5m/s);
- Airborne particle counter ARTI HHPC-6 (Hach Lange, Germany) with 6 particle count sizes of 0.3 µm; 0.5 µm; 0.7 µm; 1.0 µm; 2.0 µm; 5.0 µm and counting efficiency of 50% @0.3 µm; 100% for particles >0.45 µm (per JIS B9921:1997).

These measurement parameters were selected according to their importance for verifying the relevant ISO/DIN requirements and for their feasibility in this low resource context.

To study the microbial burden in the OT, a prospective evaluation was carried out at the end of May 2024, in a time slot when no surgical procedures were planned, a passive sampling with settling plates was carried out. The technique of passive sampling was chosen according to previous studies [10], [18]. The settling plates were placed in the OT at the locations where asepsis would be required during surgery: At the operating table (6 plates, simulating the wounds) as well as at the nurse’s table and at the instrument tables (4 plates, simulating the surgical instruments). In total ten petri dishes of 9 cm diameter containing Muller Hinton were used per measuring time point. The Petri dishes were left open for one hour, after which they were closed and incubated for 48 hours prior to colony forming units (cfus) counting. One measurement was performed with the air filtration system deactivated, another was performed with the air filtration system in use (just after the activation of the system). 

### Design and construction of the air filtration system

A simple and robust design was chosen for the air filtration system, aligned as closely as possible to DIN 1946-4. The system was designed to be realised without any significant changes to the building structure and at low cost, given the conditions. At the same time, as many components as possible should come from the hospital’s local environment. 

When choosing the filter design, it was important that the filters

1. are available everywhere (standard filters);
2. are easy to handle (pre-filter stages, compact filters) and
3. have large filter surfaces and therefore long service lives. We made a conscious decision not to comply with VDI 6022 which includes hygiene regulations for changing filters after one year.

We additionally protected the EN 1822 H13 (HEPA) filters with handle protection on both sides: This enables less qualified personnel to change the filters. Care was taken to ensure that the filters were designed appropriately, in particular 4x EN 1822 H13 filters at 3,500 m³/h, which enables the intended long service life.

We considered wood, metal, plastic and walls as possible materials for pipes. However, the prerequisite is that the pipe system has a smooth surface. We opted for plastic pipes because they were inexpensive, readily available, and met the requirements.

The type of filter housing selected depends on the locally available materials; we chose sheet steel. The wall thickness of the sheet metal is of secondary importance. The stability of the housing must be ensured, potentially through reinforcements on the outside.

**Figure 1:**
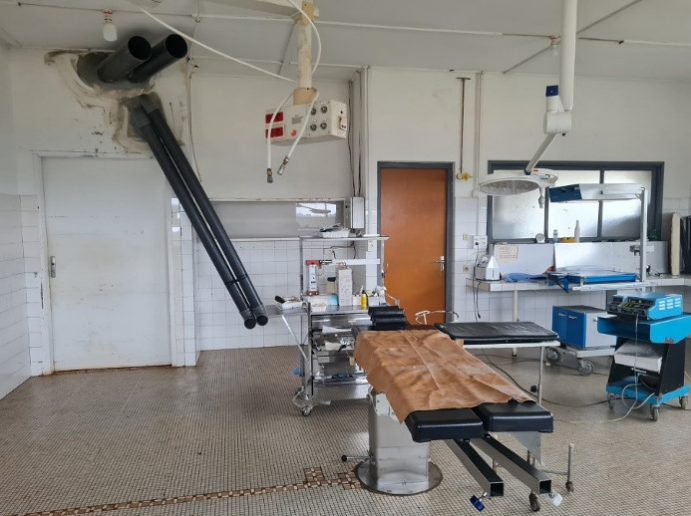
Large operation theatre

The fan’s volume flow should be between 3,000 and 3,500 m³/h, as calculated for the chosen design and filters. This amount of air guarantees the required air exchange in the operating room in our setting: The Mbouo Protestant Hospital has two operating rooms with a room height of 3.3 m; one large room 7.5 m x 7.1 m (volume approx. 176 m³), see Figure 1 (Fig. 1); one small room 6.1 m x 5.1m (approx. 103 m³).

We distributed the air to the two operating rooms using air-conducting pipes: the ratio of the large room to the small room was 2:1, as was the ratio of the return air. 

Due to the low-pressure curves of our selected filters, it was not necessary to choose a regulated fan.

**Figure 2:**
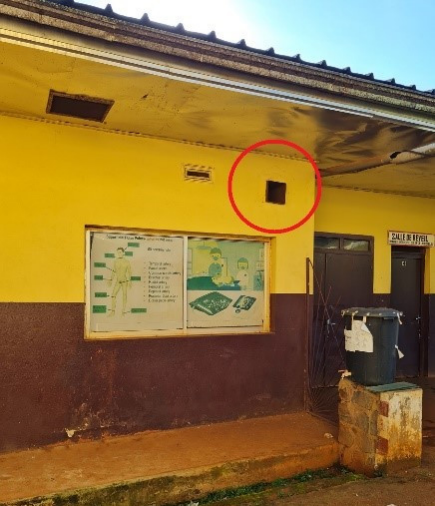
Outside fresh air collector (red circle)

In conclusion, we decided on the following context-adapted design: Fresh air is collected outside the OR building just underneath the roof (see Figure 2 (Fig. 2)).

**Figure 3:**
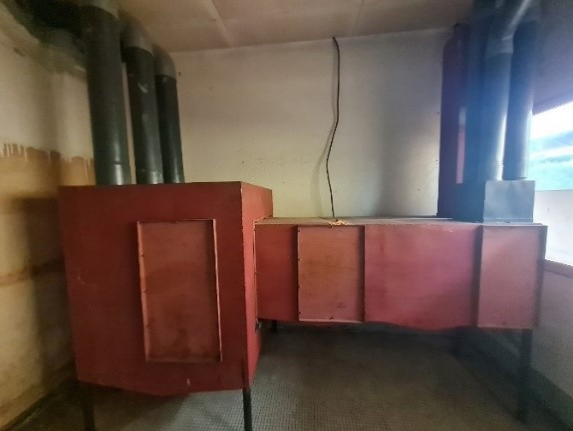
Air system box, with closed maintenance doors

**Figure 4:**
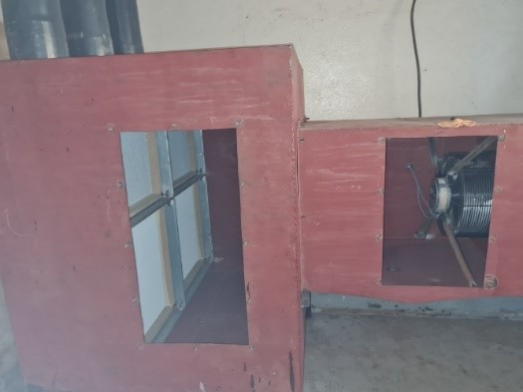
Air system box, with open maintenance doors; H13 filters and radial ventilator visible

**Figure 5:**
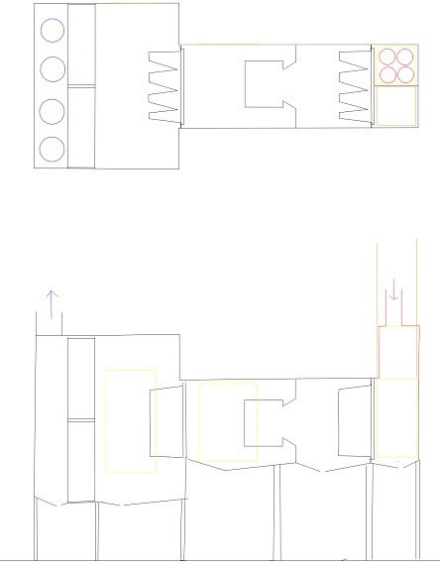
Schema of the air system box, view from above (up) and from the side (down)

This air arrives at the air system box where it is mixed with return air from the OT. It then passes a filter ISO 16890 ePM1 55% (Opakfil Energy Saver F7; CAMFIL, Germany) before arriving at the ventilator. A radial ventilator R3G355-AI56-01 (EBM-Papst, Germany) is in use, it has a volume flow of about 3,500 m³/h at a pressure difference of 500 Pa at around 2,200 rotations/minute, consuming around 900 W. Subsequently, the air passes subsequently through a filter ePM1 80% (Opakfil Energy Saver F9; CAMFIL, Germany) and through 4 parallel filters EN1822 H13 HEPA (MS13-2G10; CAMFIL, Germany; see Figure 3 (Fig. 3), Figure 4 (Fig. 4) and Figure 5 (Fig. 5) for the actual air system box and the construction schema). 

Then clean air is distributed to the OT by three PVC pipes of around 20 cm diameter each, two pipes arriving at the big OT and one pipe arriving at the small OT. The air in the OT is distributed by turbulent mixing airflow. The return air is collected by four PVC pipes with a smaller diameter (approximately 10 cm): two return pipes from the big OT, one from the small OT and another one from the foyer. 

A local welder from Bafoussam carried out the construction from January to May 2019. The system was installed in the OT at Mbouo Protestant Hospital in May and June 2019. 

The system was first put in use in June 2019 and is in regular use since that date. 

Total production and installation cost was around 3,500 €: Filters and filter boxes of CAMFIL (1,754 €), metal material and welding (673 €), pipes (680 €), ventilator (212 €) and electronics/cables (20 €).

### Data processing and statistical analysis

All data from the maintenance measurements and microbial testing were collected in an Excel file (Microsoft Corporation). The average particle count per litre air was calculated as the mean of 3 measurements at the same time point. The mean quantity of bacterial cfus was calculated out of the 10 settling plates per time point. The statistical analysis was done with R (R foundation for statistical computing). For statistical analysis regarding the cfus, the t-test and confidence intervals were calculated. Two-tailed p-values were calculated and p-values of <0.05 were considered statistically significant. 

### Ethical considerations

As this was a study without any patient contact during the prospective and retrospective parts, no informed consent of patients was necessary to consider. The study was approved by the Cameroon Regional Ethics Committee (Comité d’Ethique Régional pour la Recherche en Santé Humaine de la Region de l’Ouest CRESH-OU; Address: B.P. 479 Bafoussam, Cameroon; email: ethiqueouest@gmail.com), reference No. 503/29/05/2024/CE/CRERSH-OU, issued 29^th^ May 2024. The study is registered at clinicaltrials.gov with ID NCT07034573.

## Results

### Particle counts of the air

At the air inlets of both the small and the large operating room, we found a particle count of 0 at all measurement time points and particle sizes, so the requirements according to DIN1946-4 have been met in this regard (Table 1 (Tab. 1)).

Before starting the ventilation system, the particle count in the operating theatres were quite high, similar to the air outside the OT building. One hour after switching on the ventilation system, a significant air purification in the OT was achieved with a reduction of >98% in the quantity of particles from 0.3 to 2 µm. For particles of 5 µm, a reduction of >82% was achieved after one hour and >97% after three hours.

The results were similar, even a little better for the small operating room. The small operating room achieved clean room class 6 according to ISO 14644-1 in the area over the operating table and instrument areas; the large operating room nearly achieved it.

### Microbiological contamination

When the air filtration system was deactivated (control group) — meaning the OT had ‘natural ventilation’ — a mean bacterial growth of 17.1 cfus per settling plate was observed. In the intervention group, settling plates opened just after the air filtration system was activated showed a mean growth of only 4.8 cfus. 

This difference corresponds to a reduction of the microbial burden in the OT air by 72% with the activation of the air filtration system. The difference is highly significant (p<0.001). 

The agar plates showed predominantly Gram-positive bacterial growth (staphylococci) and some Gram-negative bacteria (Enterobacteriaceae).

### Technical evaluation of the filtration system

The air filtrations system has been in regular use since its installation in June 2019. Due to COVID travel restrictions, the first maintenance could only be performed with quite a delay in November 2023. However, after 4 years of regular use, the system still worked without malfunction. The following measurements are from this maintenance:

- The differential pressure of all the filters was still in the acceptable range. The Opakfil Energy Saver F7 showed an increase in differential pressure from 65 Delta-Pa to only 120 Delta-Pa over the 4 years. The Opakfil Energy Saver F9 and the H13 filters showed no relevant increase in differential pressure. These relatively low increases in differential pressure indicate that the filters are still functioning well, meaning that their service life is longer than four years in our case.
- The total volume flow at the two air inlets of the large operating room was about 1,700 m³/h, at the small operating room around 870 m³/h, which corresponds to a total volume flow of the ventilation system of approximately 2,500 m³/h. The airflow velocity at the air inlets was approximately 7.4 m/s.
- The air return from the large operating room was around 260 m³/h in total, by 2 air return pipes. One other air return pipe was in the small operating room, another one in the foyer between the operating rooms.
- During the morning and evening hours, the temperature in the operating rooms without the ventilation system in use ranged mostly from 18 to 20°C, with relative humidity between 87 and 94%. At noon, the temperature was around 23–25°C with a relative humidity of 60–65%. When activating the ventilation system, it accelerated the fast increase in temperature in the morning to values around 24 to 27°C and decrease in humidity of around 55%. Activating the ventilation system at noon and in the evening had no significant effect on temperature or humidity. Of course, these values depend on the outside air conditions, as the ventilation system is not equipped with a climate control unit.
- The operating rooms showed a positive differential pressure to the foyer room between +6 Delta-Pa (large operating room – foyer) and +4 Delta-Pa (small operating room – foyer). Taking the volume flow and the volume of the operating rooms into account, the ventilation system ensures between 8.4 (small operating room) and 9.6 (large operating room) air changes per hour.

Some important DIN 1946-4 requirements for class Ib operating rooms are met, like air cleanliness DIN EN ISO 14644-1 class 5 at the air inlet, a positive differential pressure from the operating room to the neighbouring room, a three stage filtration and an outside air proportion of >1,200m³/h. Other requirements of DIN 1946-4 are not completely met (Table 2 (Tab. 2)).

## Discussion

There existed initiatives to design low cost air filtration ventilation systems, but only for high-income countries [19]. This pilot study is, to our knowledge, the first of its kind studying the effectiveness of an affordable high-efficient context-adapted mechanical air filtration system for OT in a low resource setting.

Many of the aims of the development were met during the construction and implementation:

- A large part of the air filtration system was built locally with locally available materials (estimated around 70%). This strengthens local economy, lowers constructions prices and facilitates local maintenance and modifications.
- The system had no malfunction even in the absence of maintenance. This is a critical issue, as infrequent or absent maintenance often leads to the premature failure of medical equipment in low resource environments. Biomedical maintenance technicians and spare parts are sometimes scarce. A robust equipment which requires only minimal maintenance may be the best option for these contexts. The choice for large filter surfaces with a long service life of more than 4 years underlines that point. A simple design without microelectronics (except the ventilator) proved its robustness in the face of frequent power failures and voltage fluctuations regularly happening in this environment.
- The system was easy to handle by the personnel. An on-off-rotary potentiometer was the only control element.

The technical evaluation showed that this air filtration system is not fully ISO/DIN [12], [13], [14], [15] compliant (see Table 2 (Tab. 2)). But, according to our data, the system is affordable and works in this low resource context. It reduces the airborne particles in the operating room by >97% and – even more important in our opinion – lowers bacterial growth on settling plates by 72%. The latter seems the most important measurement criterion concerning infection prevention, as it simulates the ‘settling’ of bacteria from the air in the surgical site wound and on the surgical instruments and implants. It provides thus a relevant improvement to the former window-based ‘natural’ ventilation.

The application of strict ISO/DIN standards to settings with limited resources is a sensitive topic that requires critical discussion. However, this topic exceeds the scope of this article. The authors strongly believe that challenging environments require context-adapted solutions to achieve the best possible outcomes for patients with limited resources; that is also the unreserved opinion of the editor-in-chief.

This proposed air filtration system seems to be a cost-efficient (3,500 € instead of >50,000 €) and context-adapted alternative to the often used “simple air conditioning systems” or “window-based ventilation” in OT in low resource settings. The installation cost is around 3,500 € and the annual operating costs of our proposed system are estimated around 300 € (electricity and spare parts).

When comparing the results of our study with other studies, we found several publications regarding particle counts and microbial air contamination in OT in – at least partly – comparable settings. Pereira et al. [9] measured the particle concentration in OT in Brazil that were ventilated with conventional wall-mounted air conditioning systems; he found an average total particle concentration of 22.1 particles/cm³ which was about 100 times higher than the total particle concentration we found with activated ventilation system, which was 0.18 particles/cm³. In contrast to that, we found a total particle concentration of 64.3 particles/cm³ in our study when the ventilation system was deactivated, which was about 3 times higher than the particle concentration in Brazil.

Regarding the microbial air contamination in OT, we could identify several studies measuring the microbial contamination with similar methods as we used in our study (settlement plates open for one hour; incubation for 48 hours, diameter of settlement plates 9 cm). Hirsch et al. [10] compared four different ventilation systems in Germany: window-based ventilation, supported air nozzle canopy, low-turbulence displacement air flow with and without airflow stabilizer; a mean bacterial burden of 13.3 cfus was found for window-based ventilation, of 6.4 cfus for supported air nozzle canopy, of 3.4 cfus for low-turbulence displacement airflow without stabilizer and of 0.8 cfus with stabilizer. These findings are in the range of the results obtained by our study with a bacterial burden of 17.1 cfus for deactivated ventilation system (comparable to window based ventilation) and of 4.8 cfus for activated ventilation system (comparable to supported air nozzle canopy). When comparing these results, it is important to note that, unlike in our study, Hirsch et al. [10] took the measurements during ongoing surgery and not in an empty OT. This could have had a negative impact on the bacterial burden, resulting in higher CFU counts.

Another study by Montagna et al. [18] showed bacterial burden of median 4.5 cfus during an operation and median 0 cfu without operation in OT in Southern Italy, with similar results for turbulent and mixing airflow systems.

Pasquarella et al. [20] compared different ventilation systems of Italian hospitals in terms of bacterial burden in OT air during arthroplasty operations and SSI. For turbulent airflow systems (like in our study), a mean bacterial burden of 9.7 cfus was found, whereas unidirectional airflow systems showed a lower burden (mean 5.4 cfus) and mixed airflow systems showed a higher burden (17.4 cfus). No relevant statistically significant differences were found in terms of SSI between the different systems. 

A study in OT in Ghana [21] using non-laminar airflow system with HEPA filters showed bacterial burden in the OT air exceeding recommended values in 51% of cases in empty OT, indicating a need for measures to improve the ventilation, like regular maintenance of HEPA filters. During surgery, the bacterial burden in OT air exceeded the recommended limits in 84% of cases, high burden being correlated to door openings and personnel present. Reducing door opening during surgery and minimizing the personnel present during surgery could be further improve bacterial burden during operations. 

A study performed by Thomas et al. in Malawi [22] in an OT using natural ventilation showed a bacterial burden of 8 cfus near the operating table without operation. Although this value is lower than the 17 cfus reported in our study, the agar plate exposure time was only 30 minutes — half that of our study — and the incubation time was likewise halved.

Another study from the Hawassa University Referral Hospital in Ethiopia [23] found a mean bacterial load of 8.6 cfus/dm² in OT at rest, which is higher than the 7.6 cfus/dm² in our study. However, this study did not state which ventilation system was used in their OT.

Other possible solutions for affordable ventilation systems adapted to the context of low resource settings may be the ‘upflow displacement’ ventilation system [24] or a low-cost low-turbulence displacement-flow ventilation system [25]. To our knowledge, these two alternatives have not yet been implemented and tested in low-resource settings.

In addition to the discussion on the most suitable technical solution for air cleanliness in the OT, it should be emphasised that discipline plays an important role in asepsis [26]. SSI prevention is best achieved by implementing all the different measures set out in the international guideline for the prevention of SSI [3].

### Limitations

The main limitations of the present study are its implementation in only one hospital and its observation period of only four years. As this was a pilot study, further scaling up and long-term evaluations are required.

The present study did not measure the effect of the ventilation system on reducing SSIs. This was beyond the scope of the present study, but should be the focus of future intervention studies. However, a recently published observational study of SSIs at the study site (Mbouo Protestant Hospital) showed a relatively low SSI incidence compared to the Sub-Saharan Africa context [27]. One possible contributing factor is the implemented air ventilation system, which may indicate a beneficial effect in reducing SSIs.

### Strengths

This study presents several strengths:

1. It is the first study of its kind regarding the implementation and evaluation of an affordable air ventilation system for OT in a low resource setting, to our knowledge.
2. The implementation phase is already completed, so the ventilation system was already tested in “real context conditions”.
3. Many technical measurements were made and are published here, so discussion and improvements of the ventilation system should be facilitated.
4. The construction plans of the ventilation system are freely available (see https://www.apavent.de/).
5. This is one of the rare but urgently needed studies regarding OT air in low and middle income countries [21].
6. The proposed ventilation system reduce the bacterial load in the OT air in a cost-effective, low-maintenance, locally produced manner.

### Outlook

Based on the results of this pilot study, further studies are needed, focusing on a longer follow-up period on this hospital and a scaling up to others hospitals of similar low resource contexts. The scaling up to other hospitals could even be planned as interventional studies to measure the effect on SSI against natural ventilation/simple air conditioning. A qualitative investigation on the perceptions of personnel and patients regarding the ventilation system seems also beneficial to tailor the system even better to the local needs.

Another important need seems the discussion on standards for operating rooms in low resource settings: Should the norms and standards of high-resource settings be applied to low-resource settings without adaptation? Or do we need new, context-specific ventilation system norms for low-resource settings?

## Conclusions

This pilot study showed the feasibility and quality of a new context-adapted affordable air filtration system for OT in a low resource setting. It greatly reduces the microbial burden in the OT air, even if not all of the ISO/DIN specifications are fully met. The construction plans are freely available. Further studies and upscaling are needed, as well as a discussion on context-adapted OT air norms for low resource settings.

## Notes

### Competing interests

The authors declare that they have no competing interests.

### Ethical approval 

The study was approved by the Cameroon Regional Ethics Committee (reference No: 503/29/05/2024/CE/CRERSH-OU, issued 29^th^ May 2024). The study is registered at clinicaltrials.gov with ID NCT07034573.

### Funding

None. 

### Acknowledgments

The authors thank the personnel of the Protestant Hospital Mbouo for their assistance in collecting the data. We also thank the personnel of the Evangelical University Institute of Cameroon for their cooperation. A special thank goes to CAMFIL for their support and donation of material. We thank the Charité University Medical Centre Berlin, the German Hospital Partnerships program, the German charity organization “Paix et Santé – Gesundheit und Frieden global e.V.” and other involved national and international partners for their cooperation.

### Authors’ ORCIDs

- Doll C: https://orcid.org/0000-0001-5956-890X
- Opalka R: https://orcid.org/0009-0002-0079-4341
- Trampuz A: https://orcid.org/0000-0002-5219-2521

## Figures and Tables

**Table 1:**
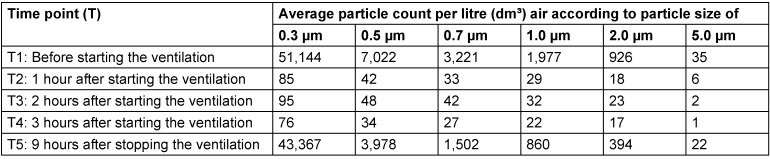
Particle counts according to particle sizes, measured over the operating table/instrument area in the large operating room, at time points 1 to 5

**Table 2:**
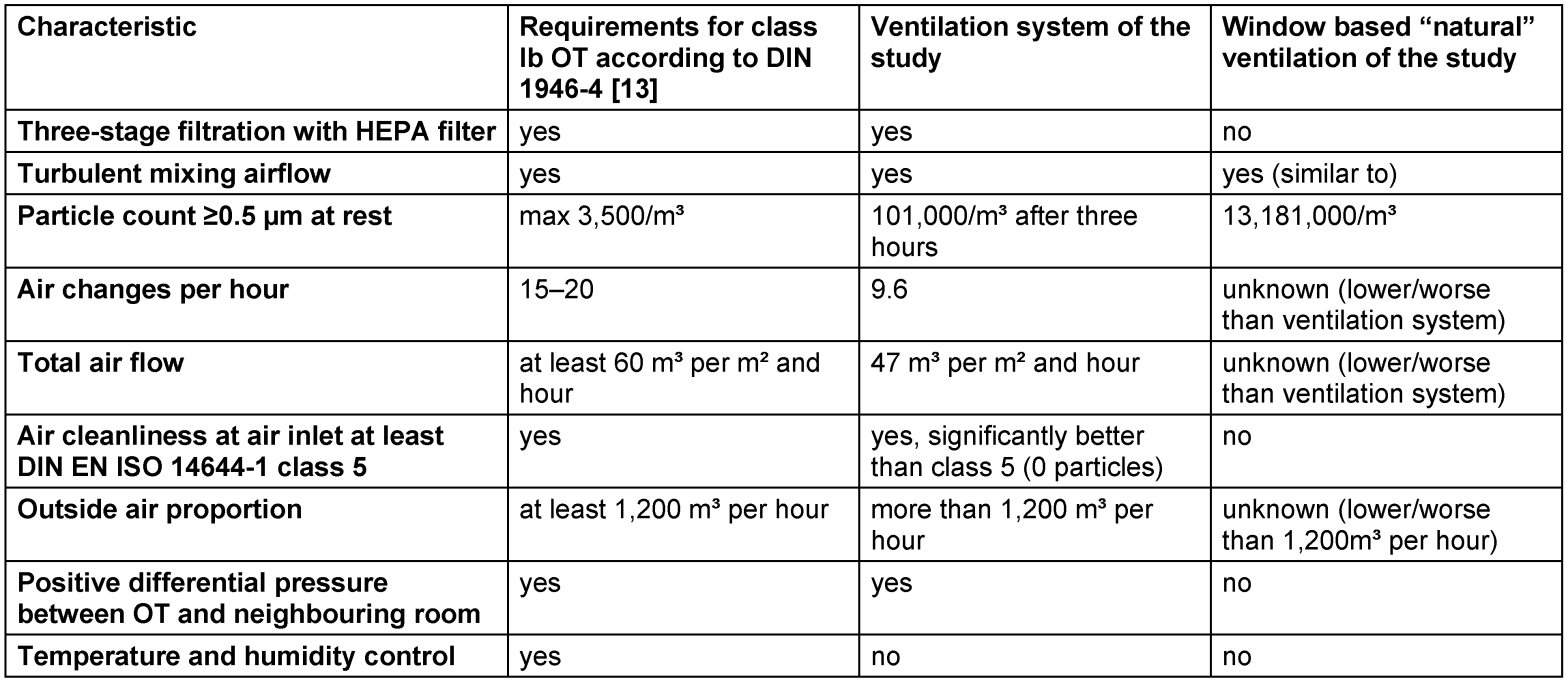
Technical data of the ventilation system of the present study, in comparison to selected DIN 1946-4 requirements and to the window-based ventilation

## References

[1] Allegranzi B, Bagheri Nejad S, Combescure C, Graafmans W, Attar H, Donaldson L, Pittet D (2011). Burden of endemic health-care-associated infection in developing countries: systematic review and meta-analysis. Lancet.

[2] Bagheri Nejad S, Allegranzi B, Syed SB, Ellis B, Pittet D (2011). Health-care-associated infection in Africa: a systematic review. Bull World Health Organ.

[3] (2018). Global Guidelines for the Prevention of Surgical Site Infection.

[4] Charnley J (1972). Postoperative infection after total hip replacement with special reference to air contamination in the operating room. Clin Orthop Relat Res.

[5] Nwankwo E, Edino S (2014). Seasonal variation and risk factors associated with surgical site infection rate in Kano, Nigeria. Turk J Med Sci.

[6] Sadrizadeh S, Aganovic A, Bogdan A, Wang C, Afshari A, Hartmann A (2021). A systematic review of operating room ventilation. J Build Eng.

[7] Evans RP (2011). Current concepts for clean air and total joint arthroplasty: laminar airflow and ultraviolet radiation: a systematic review. Clin Orthop Relat Res.

[8] Zhang B, Li L, Yao X, Gong Y, Zhang Y, Yang H, Li W, Lin L, Yang Y, Zhang H, Jia H (2020). Analysis of Air Purification Methods in Operating Rooms of Chinese Hospitals. Biomed Res Int.

[9] Pereira M, Tribess A, Buonanno G, Stabile L, Scungio M, Baffo I (2020). Particle and Carbon Dioxide Concentration Levels in a Surgical Room Conditioned with a Window/Wall Air-conditioning System. Int J Environ Res Public Health.

[10] Hirsch T, Hubert H, Fischer S, Lahmer A, Lehnhardt M, Steinau HU, Steinstraesser L, Seipp HM (2012). Bacterial burden in the operating room: impact of airflow systems. Am J Infect Control.

[11] Boppre D, Exner M, Krüger CM, Schuler H, Wendt M, Harnoss JC, Kramer A (2024). Achieving room air quality of room class Ib in the aseptic area using a mobile sterile ventilation unit in a room class II surgical unit. GMS Hyg Infect Control.

[12] Külpmann R, Christiansen B, Kramer A, Lüderitz P, Pitten FA, Wille F, Zastrow KD, Lemm F, Sommer R, Halabi M (2016). Hygiene guideline for the planning, installation, and operation of ventilation and air-conditioning systems in health-care settings – Guideline of the German Society for Hospital Hygiene (DGKH). GMS Hyg Infect Control.

[13] (2018). DIN 1946-4:2018-09, Raumlufttechnik - Teil 4: Raumlufttechnische Anlagen in Gebäuden und Räumen des Gesundheitswesens.

[14] (2016). DIN EN ISO 14644-1:2016-06 Reinräume und zugehörige Reinraumbereiche - Teil 1: Klassifizierung der Luftreinheit anhand der Partikelkonzentration (ISO 14644-1:2015).

[15] (2018). VDI 6022 Blatt 1:2018-01 Raumlufttechnik, Raumluftqualität - Hygieneanforderungen an raumlufttechnische Anlagen und Geräte (VDI-Lüftungsregeln).

[16] (2017). DIN EN ISO 16890-1:2017-08 Luftfilter für die allgemeine Raumlufttechnik - Teil 1: Technische Bestimmungen, Anforderungen und Effizienzklassifizierungssystem, basierend auf dem Feinstaubabscheidegrad (ePM) (ISO 16890-1:2016); Deutsche Fassung EN ISO 16890-1:2016.

[17] (2019). DIN EN 1822-1:2019-10 Schwebstofffilter (EPA, HEPA und ULPA) - Teil 1: Klassifikation, Leistungsprüfung, Kennzeichnung; Deutsche Fassung EN 1822-1:2019.

[18] Montagna MT, Rutigliano S, Trerotoli P, Napoli C, Apollonio F, D'Amico A, De Giglio O, Diella G, Lopuzzo M, Marzella A, Mascipinto S, Pousis C, Albertini R, Pasquarella C, D'Alessandro D, Serio G, Caggiano G (2019). Evaluation of Air Contamination in Orthopaedic Operating Theatres in Hospitals in Southern Italy: The IMPACT Project. Int J Environ Res Public Health.

[19] Minns RJ, Flynn M, Ljunggren AH, Agnew JC (1979). Design and evaluation of a low cost columnar flow ventilation scheme in an operating theatre. Eng Med.

[20] Pasquarella C, Barchitta M, D'Alessandro M (2018). Heating, ventilation and air conditioning (HVAC) system, microbial air contamination and surgical site infection in hip and knee arthroplasties: the GISIO-SItI Ischia study. Ann Ig Med Prev E Comunità.

[21] Stauning MT, Bediako-Bowan A, Andersen LP, Opintan JA, Labi AK, Kurtzhals JAL, Bjerrum S (2018). Traffic flow and microbial air contamination in operating rooms at a major teaching hospital in Ghana. J Hosp Infect.

[22] Thomas S, Palmer R, Phillipo E, Chipungu G (2016). Reducing bacterial contamination in an Orthopedic Theatre ventilated by natural ventilation, in a Developing Country. J Infect Dev Ctries.

[23] Mengistu H, Misganaw B, Elshaday A (2016). Bacterial load and antibiotic susceptibility pattern of isolates in operating rooms at Hawassa University Referral Hospital, southern Ethiopia. J Microbiol Antimicrob.

[24] Short CA, Woods AW, Drumright L, Zia R, Mingotti N (2022). An alternative approach to delivering safe, sustainable surgical theatre environments. Build Cities.

[25] Heinz J, Schäfer MB, Stewart KW, Pott PP (2019). Low-turbulence displacement-flow for an operating environment: Specifications, prototyping and first measurements of a low-cost solution. Curr Dir Biomed Eng.

[26] Popp W, Alefelder C, Bauer S, Daeschlein G, Geistberger P, Gleich S, Herr C, Hübner NO, Jatzwauk L, Kohnen W, Külpmann R, Lemm F, Loczenski B, Spors J, Walger P, Wehrl M, Zastrow KD, Exner M (2019). Air quality in the operating room: Surgical site infections, HVAC systems and discipline – position paper of the German Society of Hospital Hygiene (DGKH). GMS Hyg Infect Control.

[27] Doll C, Ndoho Simo LC, Jeulefack H, Tamuedjoun Talom A, Kuate Kamdem L, Kenmogne JB, Djeunang Dongho GB, Trampuz A (2025). Efforts in surgical site infection surveillance at the Mbouo Protestant Hospital in Cameroon. BMC Surg.

